# Plastome evolution and organisation in the *Hoya* group (Apocynaceae)

**DOI:** 10.1038/s41598-021-93890-6

**Published:** 2021-07-15

**Authors:** Michele Rodda, Matti A. Niissalo

**Affiliations:** Singapore Botanic Gardens, National Parks Board, 1 Cluny Road, Singapore, 259569 Singapore

**Keywords:** Plant evolution, Plant genetics

## Abstract

The genus *Hoya* is highly diverse and many of its species are popular ornamental plants. However, the relationships between *Hoya* and related genera (the *Hoya* group) are not fully resolved. In this study, we report 20 newly sequenced plastomes of species in the *Hoya* group. The complete plastomes vary in length from 175,405 to 178,525 bp while the LSCs vary from 90,248 to 92,364 bp and the complete SSCs vary from 2,285 to 2,304 bp, making the SSC in the *Hoya* group one of the shortest known in the angiosperms. The plastome structure in the *Hoya* group is characterised by a massive increase in the size of the inverted repeats as compared to the outgroups. In all ingroup species, the IR/SSC boundary moved from *ycf1* to *ndhF* while this was not observed in outgroup taxa, making it a synapomorphy for the *Hoya* group. We have also assembled the mitogenome of *Hoya lithophytica*, which, at 718,734 bp, is the longest reported in the family. The phylogenetic analysis using exons from 42 taxa in the *Hoya* group and three outgoups confirms that the earliest divergent genus in the *Hoya* group is *Papuahoya*, followed by *Dischidia*. The relationship between *Dischidia* and the clade which includes all *Hoya* and *Oreosparte* taxa, is not fully supported. *Oreosparte* is nested in *Hoya* making it paraphyletic unless *Clemensiella* is recognised as a separate genus.

## Introduction

*Hoya* R.Br., with 350–450 species^1^ is the largest genus in Apocynaceae-Asclepiadoideae-Marsdenieae, and the second largest genus in Apocynaceae after *Ceropegia* L.^2^. It includes epiphytic or more rarely terrestrial and hemi-epiphytic vines and shrubs with leaves ranging from coriaceous to very thick and succulent. The distribution area spans from the Himalayan foothills to the northwest, Okinawa (Japan) to the northeast, Australia to the south and the Fiji Islands to the southeast. *Hoya* and the similar genera *Absolmsia* Kuntze (1 sp.), *Anatropanthus* Schltr. (1 sp.), *Clemensiella* Schltr. (2 spp.), *Dischidia* R.Br. (ca. 80 spp.), *Heynella* Backer (1 sp.), *Madangia* P.I.Forst., Liddle & I.M.Liddle (1 sp.), *Micholitzia* N.E.Br. (1 sp.), and *Oreosparte* Schltr. (3 spp.), have been generally called the “*Hoya* group”^3–6^. *Absolmsia*, *Anatropanthus*, *Clemensiella*, *Micholitzia*, as well as *Eriostemma* (Schltr.) Kloppenb. & Gilding and *Hiepia* V.T.Pham & Aver., have been subsumed under *Hoya* based on molecular data^7–10^ and therefore at present the *Hoya* group includes only *Dischidia*, *Heynella* (for which no molecular data is available), *Hoya*, and the recently published *Papuahoya* Rodda & Simonsson (3 species)^7^.

The best sampled analysis of the morphological and taxonomic diversity of the *Hoya* group conducted to date is based on three chloroplast loci (*trnT-UGU*–*trnL-UAA*–*trnF-GAA*, *psbA–trnH*, *matK*) and two nuclear loci (ITS and ETS)^7^. The *Hoya* group clade, including *Hoya s*.*l.*, *Dischidia*, *Oreosparte* and *Papuahoya*, is nested within Marsdenieae in a clade with other Asian and Australasian species. *Hoya* is paraphyletic unless *Dischidia* and *Oreosparte* are synonymised. However, the relationships between the two main *Hoya* clades, *Oreosparte* and *Dischidia* are not supported and there is no sufficient evidence to synonymise *Oreosparte* and *Dischidia* with *Hoya*. *Papuahoya*, from New Guinea, originally suspected to be part of *Oreosparte* based on morphological similarities, is sister to the rest of the *Hoya* group but only with 79% bootstrap support (BS).

Several studies have focused on evolution of plastomes in Apocynaceae, spanning much of the diversity of the family. The first complete plastomes in the family were of *Asclepias syriaca* L.^11^ and *Catharanthus roseus* (L.) G.Don^12^. These plastomes were compared to the available plastomes in Gentianales: Straub et al.^11^ reported the loss of *accD*, *clpP*, and *ycf1* in *A*. *syriaca*, and Ku et al.^12^ found that the plastome of *C*. *roseus* is highly similar to that of *Coffea arabica* L. (Rubiaceae), with no gene losses. Ku et al.^12^ noted another missing gene in *A. syriaca* compared to *C. roseus* (*ycf15*), as well as an expansion in some intergenic regions in *A*. *syriaca* and a difference in the position of inverted repeat boundaries. The plastome changes in *Asclepias* L., and the dynamics between plastomes and mitogenomes were reported in detail by Straub et al.^13^. They found evidence of gene movement from the mitogenome to the plastome, which is unusual in angiosperms^14^. Straub et al.^15^ expanded the taxonomic sampling to 12 plastomes within Apocynaceae, and concluded that plastome structure is highly conserved in the family, with the exception of the genus *Asclepias*. Fishbein et al.^16^ published 73 complete plastomes from the family, and their plastome phylogeny was used to assess taxonomic relationships within the family. Several other plastomes have also been published^17–24^. Only three plastomes of *Hoya* have been published so far. *Hoya* *pottsii* Traill (a synonym of *Hoya* *verticillata* (Vahl) G.Don) and *Hoya* *liangii* Tsiang (a synonym of *Hoya* *diversifolia* Blume)^25^ were reported to have a plastome architecture similar to that of other Apocynaceae, and *Hoya* *carnosa* (L.f.) R.Br.^26^ was reported to have a near complete loss of the small single copy regions (SSC) of the plastome due to a boundary shift leading to a large expansion of the two inverted repeats (IRs).

Mitogenomes of Apocynaceae have been less thoroughly studied. In *Asclepias* (subfamily Asclepiadoideae), Straub et al.^13^ reported substantial import of plastome regions to the mitogenomes, and major restructuring of the genomes when compared to closest relatives; both are common features in plant mitogenomes. Park et al.^27^ similarly reported in *Rhazya* Decne. (informal group Rauvolfioids) repeat regions and movement of genetic material from the nucleus and plastome to the mitogenome, but no movement from the mitogenome to plastome. In *Cynanchum* L. (subfamily Asclepiadoideae) the mitogenome is reported to be multipartite, consisting of two chromosomes^28^.

In this paper, we sequenced and assembled the complete or near complete plastomes of 20 species in the *Hoya* group. Our aim was to investigate the evolutionary position of the structural changes reported by Wei et al.^26^ with a broader sampling of taxa. In addition, we assembled the plastome exons of a larger number of species (39) to provide maximum support for a phylogenetic reconstruction of the evolution of plastomes in this group. This complete plastome phylogeny (omitting only poorly aligning intergenic areas and very short exons) will be an invaluable resource when interpreting nuclear phylogenies in the group, and will provide a backbone against which reticulation events and poorly resolved trees can be compared.

## Results

### Plastome structure in the *Hoya* group

We acquired complete plastomes for ten species in the ingroup and two in the outgroup. For a further ten species in the ingroup, we acquired near complete plastomes, with 1–6 gaps in mononucleotide regions with low coverage (Table 1). For the remaining 19 species (18 ingroup, one outgroup), all targeted exons were acquired.

**Table 1:** Summary of 22 plastomes (12 complete plastomes) of 4 species of *Dischidia*, 14 of *Hoya*, 1 of *Oreosparte* and 1 of *Papuahoya* (ingroups), and two outgroups.

Sample	Length of plastome	Length of LSC	Length of SSC	Length of IRs	CDSs	Unique CDSs	rRNAs	unique rRNAs	tRNAs	unique tRNAs	Number of gaps	Gaps	*accD*	*ycf1*	*ycf2*	genes at IR–LSC junction	genes at IR–SSC junction	Type of material	Percentage of sequencing reads mapping to plastome
*Dischidia acutifolia*			2300	41,748	99	81	8	4	38	30	2	LSC: trnS-GCU–trnG-UCC intergenic region, clpP intron	1935	5916	6486	*rpl22*	*ndhF*	fresh	13.94
*Dischidia milnei*	175,405	90,564	2297	41,272	99	81	8	4	38	30	0		1653	5721	6480	*rpl22*	*ndhF*	fresh	16.99
*Dischidia nummularia*			2293	41,300	99	81	8	4	38	30	2	LSC: clpP intron, trnSGCU–trnG-UCC intergenic region	1659	5745	6486	*rpl22*	*ndhF*	fresh	9.80
*Dischidia parasita*	177,089	91,020	2297	41,886	99	81	8	4	38	30	0		1980	6021	6546	*rpl22*	*ndhF*	fresh	18.13
*Hoya coronaria*			2293	42,055	99	81	8	4	38	30	2	LSC: rps2–rpoC2 intergenic region, psbZ–trnG-GCC intergenic region	2208	6072	6480	*rpl22*	*ndhF*	fresh	7.79
*Hoya diversifolia*			2285		99	81	8	4	38	30	6	LSC: trnS-GCU–trnG-UCC intergenic region, trnE-UUC–trnT-GGU intergenic region, ndhC–trnV-UAC intergenic region, clpP intron, rpl16 intron. IRs: ndhA intron	1908	5913	6402	*rpl22*	*ndhF*	fresh	3.55
*Hoya exilis*	178,244	91,806	2300	42,069	99	81	8	4	38	30	0		2121	5736	6486	*rpl22*	*ndhF*	fresh	8.53
*Hoya hamiltoniorum*			2290	41,322	99	81	8	4	38	30	2	LSC: ndhC–trnV-UAC intergenic region, clpP intron	2004	5832	6453	*rpl22*	*ndhF*	fresh	6.52
*Hoya ignorata*	175,892	90,248	2296	41,674	99	81	8	4	38	30	0		1767	6033	6549	*rpl22*	*ndhF*	fresh	11.79
*Hoya insularis*			2294	41,453	99	81	8	4	38	30	1	LSC: trnE-UUC–trnT-GGU intergenic region	1881	5895	6453	*rpl22*	*ndhF*	fresh	5.36
*Hoya latifolia*			2294	41,307	99	81	8	4	38	30	2	LSC: clpP intron, trnS-GCU–trnG-UCC intergenic region	1878	5832	6426	*rpl22*	*ndhF*	fresh	6.59
*Hoya lithophytica*	176,580	90,805	2297	41,739	99	81	8	4	38	30	0		2031	5997	6486	*rpl22*	*ndhF*	fresh	21.04
*Hoya lyi*	176,567	91,623	2294	41,325	99	81	8	4	38	30	0		1767	5721	6425	*rpl22*	*ndhF*	fresh	13.63
*Hoya megalaster*	178,169	92,364	2303	41,751	99	81	8	4	38	30	0		1998	4218	6480	*rpl22*	*ndhF*	fresh	20.56
*Hoya monetteae*	176,921	91,592	2301	41,514	99	81	8	4	38	30	0		1827	5928	6435	*rpl22*	*ndhF*	fresh	15.38
*Hoya omlorii*	178,525	92,141	2304	42,040	99	81	8	4	38	30	0		1887	6279	6480	*rpl22*	*ndhF*	fresh	10.76
*Hoya thailandica*			2299	41,689	99	81	8	4	38	30	2	LSC: trnS-GCU–trnG-UCC intergenic region, clpP intron	2145	5985	6480	*rpl22*	*ndhF*	fresh	14.05
*Hoya verticillata*			2294	41,369	99	81	8	4	38	30	5	LSC: trnS-GCU–trnG-UCC intergenic region, trnE-UUC–trnT-GGU intergenic region, ndhC–trnV-UAC intergenic region, clpP intron, rpl16 intron	1677	5862	6444	*rpl22*	*ndhF*	fresh	5.15
*Jasminanthes maingayi*	161,660	90,533	18,553	26,287	88	81	8	4	37	30	0		1942	5850	6759	*rpl22*	*ycf1*	fresh	9.20
*Marsdenia flavescens*	161,700	91,559	17,907	26,117	87	81	8	4	37	30	0		2154	5832	6825	*rps19–rpl2* intergenic region	*ycf1*	Silica	6.46
*Oreosparte celebica*		unknown	2299	41,401	99	81	8	4	38	30	2	LSC: rps2–rpoC2 intergenic region, ndhC–trnV-UAC intergenic region	1857	5841	6465	*rpl22*	*ndhF*	Herbarium	3.79
*Papuahoya urniflora*	178,400	91,636	2298	42,233	99	81	8	4	38	30	0		1932	5697	6450	*rpl22*	*ndhF*	Silica	21.70

We observed that species in the *Hoya* group have a very large copy number of plastomes per cell: 3.55–21.70% of all sequencing reads mapped to the plastome (Table 1).

The total length of the complete plastomes varies from 175,405 bp (*Dischidia milnei* Hemsl.) to 178,525 bp (*Hoya omlorii* (Livsh. & Meve) L.Wanntorp & Meve) (161,660–161,700 bp in the outgroups) (Table 1). The length of complete IRs varies from 41,272 (*D. milnei*) to 42,069 bp (*Hoya exilis* Schltr.) (26,117–28,287 bp in the outgroups). The complete LSCs of the ingroups varies in length from 90,248 bp (*Hoya ignorata* T.B.Tran, Rodda, Simonsson & Joongku Lee) to 92,364 bp (*Hoya* *megalaster* Warb. ex K.Schum. & Lauterb.) and the complete SSCs varies from 2,285 bp (*H*. *diversifolia*) to 2,304 bp (*H*. *omlorii*), making the SSC in *Hoya* group one of the shortest known in angiosperms^29^.

**Figure 1:**
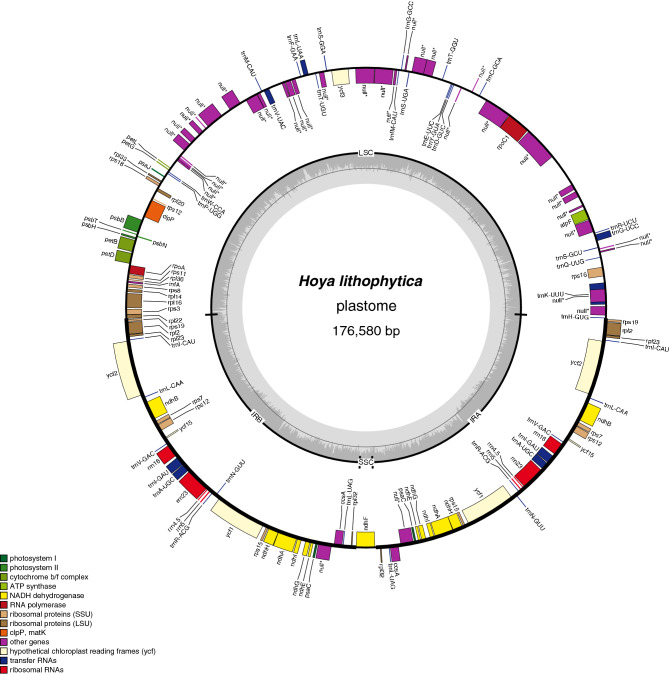
Chloroplast genome of *Hoya lithophytica*. The colour-coded bars indicate different functional groups. The darker grey area in the inner circle indicates GC content, while the lighter grey area indicates AT content. *IR* inverted repeat, *SSC* small single copy, *LSC* large single copy.

**Figure 2:**
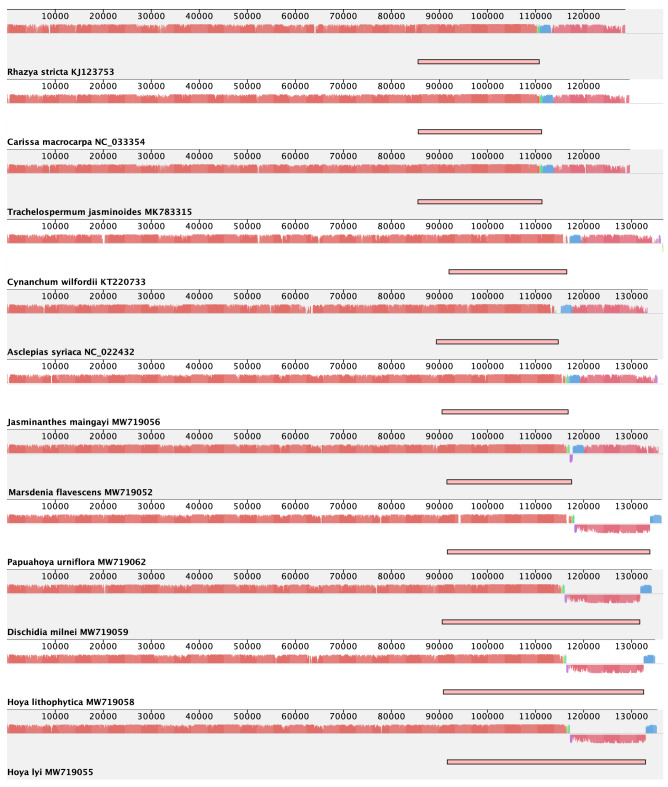
Mauve alignment of plastomes of *Hoya* group and selected other Apocynaceae species. The inverted repeat closest to *psbA* was removed, and the small single copy is displayed in a direction that best illustrates the shift in inverted repeat boundaries. The alignment colours refer to locally collinear blocks shared between plastomes. The extent of the inverted repeat is shown with a bar.

The plastome structure in all species in the *Hoya* group (Fig. 1) is characterised by a massive increase in the size of inverted repeats as compared to the outgroups; the IR/SSC boundary moved from *ycf1* to *ndhF* (Fig. 2). The outgroup species that is most closely related to the *Hoya* group, *Marsdenia flavescens* A.Cunn. ex Hook., lacks this boundary change, but it is characterised by a smaller change in the IR/LSC boundary (loss of *rps19* from IR to LSC).

**Figure 3:**
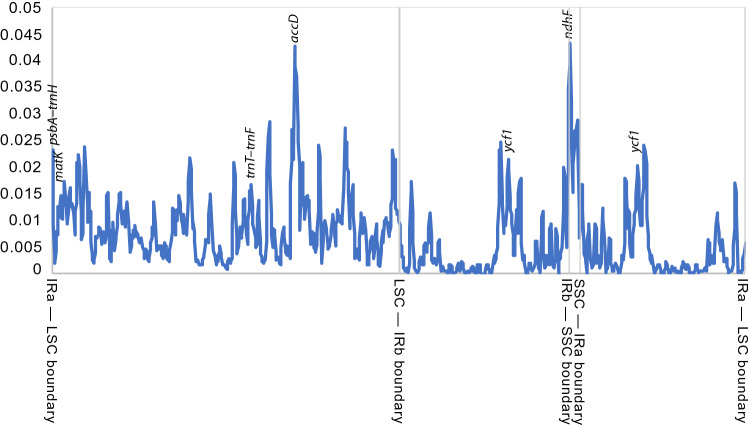
Nucleotide diversity (Pi) values of 20 complete ingroup plastomes, showing genome parts, barcoding regions commonly used in the taxa in question (*trnT-UGU*–*trnL-UAA*–*trnF-GAA*, *psbA–trnH*, *matK*) and genes in highly variable regions (*accD*, *ndhF* and *ycf1*).

The nucleotide diversity (Pi) in the ingroup varies from 0 to 0.0433 (Fig. 3). The IRs were consistently less variable than the other parts of the plastomes, except for one highly variable gene (*ycf1*). One gene in the LSC (*accD*) and the region at IR-SSC boundaries (near *ndhF*) were similarly variable. *ycf1* and *accD* were characterised by long aminoacid repeats.

### Mitogenome structure

**Figure 4:**
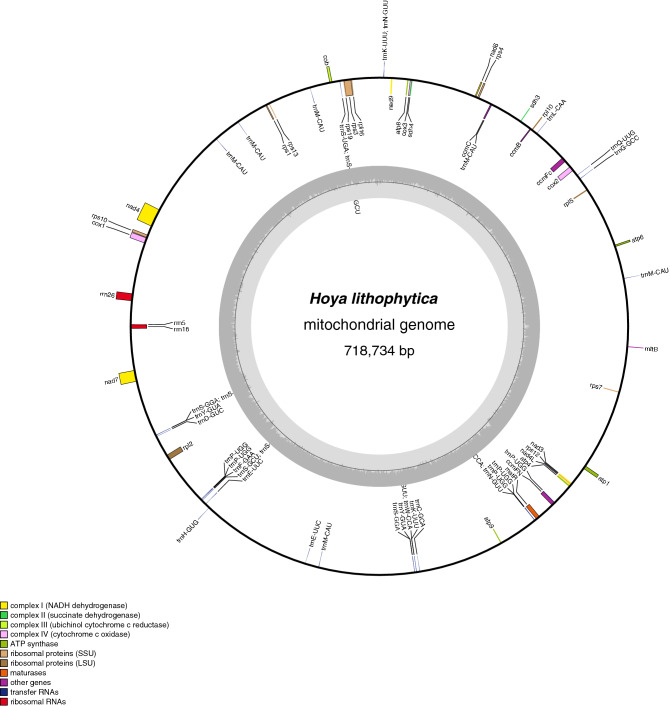
Mitochondrial genome of *Hoya lithophytica*.

**Figure 5:**
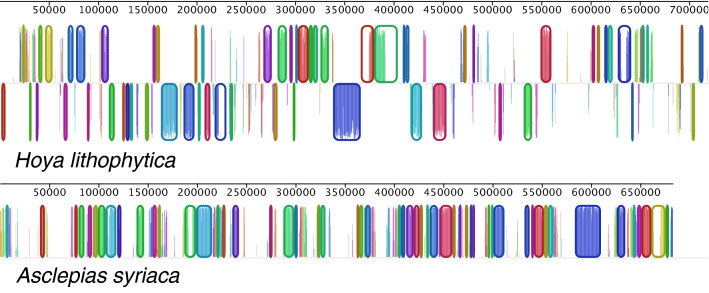
Mauve alignment of available mitogenomes in Apocynaceae, showing massive restructuring. (*Hoya lithophytica* on the top, *Asclepias syriaca* below). Corresponding blocks present in both mitogenomes are indicated by colour.

The mitogenome structure of *Hoya lithophytica* Kidyoo (Fig. 4) shows massive restructuring in relation to the other complete mitogenomes available in Apocynaceae (Fig. 5). At 718,734 bp, it is the longest mitogenome reported in the family. Movement of plastome DNA to mitogenome explains at least 56,698 bp (7.889%) of the mitogenome.

### Phylogenetic analysis

**Figure 6:**
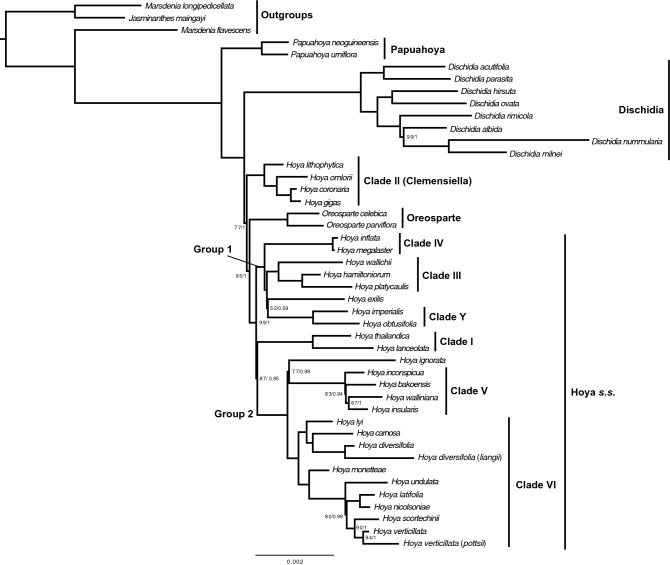
Molecular phylogeny of representative species of the *Hoya* group based on exons longer than 90 bp (excluding *accD*, *ycf1* and *ycf2*). Numbers at the nodes indicate bootstrap percentages followed by Bayesian Posterior Probability (only indicated when not fully supported).

The model choice in MrBayes had no effect on the tree topology, and only a minor effect on the node values. The two model options tested resulted in the same topology and highly similar BPP values (Bayesian Posterior Probability), differing at most by 0.05 for any node; both runs passed our quality control. The values indicated in the next paragraph and shown in the molecular phylogeny presented in Fig. 6 were acquired using GTR + Gamma.

To facilitate comparison, for the *Hoya* clades we refer to the clade names of Wanntorp et al.^10^ (their Figs. 3 and 4) and Rodda et al.^7^ (their Fig. 4) whenever possible. A new name is provided for one unidentified clade from previous studies, which includes *Hoya imperialis* Lindl. and *H*. *obtusifolia* Wight (Clade Y).

Within the *Hoya* group several well supported clades (99–100% BS, 1 BPP) can be separated. The earliest divergent clade corresponds to the genus *Papuahoya* (100% BS, 1 BPP), represented by *P*. *urniflora* (P.I.Forst.) Rodda & Simonsson and *P*. *neoguineensis* Simonsson & Rodda. The following clade includes eight species of *Dischidia* (100% BS, 1 BPP). Within *Dischidia* there is one ant-house leaved species, *D. milnei*, formerly included in the genus *Conchophyllum* Blume, that froms a clade (99% BS, 1 BPP) with the type of the genus *D*. *nummularia* R.Br. and *D*. *albida* Griff.

*Dischidia* is sister to a clade (77% BS, 1 BPP) including all *Hoya* and *Oreosparte* taxa. Within *Hoya*/*Oreosparte*, the first diverging clade includes four species of *Hoya* (100% BS, 1 BPP, Clade II) one of which was formerly classified in the genus *Clemensiella* (*C*. *omlorii, now H*. *omlorii*) and two in *Eriostemma*, [*E*. *gigas* (Schltr.) Kloppenb. & Gilding (now *H*. *gigas* Schltr.) and *E*. *coronaria* (Blume) Kloppenb. & Gilding (now *H*. *coronaria* Blume)]. These correspond to Clade II and *Clemensiella* in Wanntorp et al.^10^ and Rodda et al.^7^, respectively. Together, these three species are sister (100% BS, 1 BPP) to a recently described species of *Hoya*, *H*. *lithophytica*, from NW Thailand. Clade II is sister to a clade (95% BS, 1 BPP) including *Oreosparte* and the rest of the *Hoya* species (*Hoya s*.*s*.). *Oreosparte* (100% BS, 1 BPP) includes the type of the genus *O*. *celebica* Schltr. and *O*. *parviflora* (Ridl.) Rodda & Simonsson*.* Within *Hoya s*.*s*. (99% BS, 1 BPP) there are two larger groups (Group 1 and 2, also recognised in Wanntorp et al.^10^ and Rodda et al.^7^) and six well supported clades. Group I (100% BS, 1 BPP) is the earliest divergent group. It includes three clades: Clade IV (100% BS, 1 BPP), with two species from New Guinea; Clade III (100% BS, 1 BPP) includes three Sundaland species, two of which (*H*. *platycaulis* Simonsson & Rodda and *H*. *wallichii* (Wight) C.M.Burton) are generally non-climbing shrubs; Clade Y (not present in Wanntorp et al.^10^ and Rodda et al.^7^) includes *H*. *imperialis* and *H*. *obtusifolia*, two species from West Malesia characterised by very stout stems and a preference for sunny habitats. Sister to Group 1 is a clade (87% BS, 0.95 BPP) including Group 2 and Clade I.

Group 2 (100% BS, 1 BPP) includes two Clades: Clade V (100% BS, 1 BPP), with four species belonging to the *Hoya* section Acanthostemma; Clade VI (100% BS, 1 BPP), which includes the type of the genus, *H*. *carnosa* as well as some very widespread and variable species such as *H*. *diversifolia* and *H*. *verticillata*. Clade I (100% BS, 1 BPP), consists of two species from the Pan Himalayan area (*H*. *lanceolata* Wall. ex D.Don) and Northern Thailand (*H*. *thailandica* Thaithong).

## Discussion

With the exception of Wei et al.^26^, previously published work on plastomes of the *Hoya* group only resulted in incomplete plastomes^16,30^ or incorrectly assembled plastomes^25^. This is not surprising, as assembling plastomes in this group is very challenging: our attempts at automated sequence assembly only resulted in small fragments, which often incorporated mitogenome sequences and required extensive manual corrections. Likely causes of the difficulty in assembling the genomes are the extensive expansion of IRs, and the near-loss of SSC, the large amount of sequences shared by the plastome and the mitogenome and the frequent repetitive elements found in the plastomes.

In our experience, the fastest method of genome assembly is assembly of reads to a reference, followed by manual correction. A highly time-consuming process of manual checking of the entire alignment followed all initial assemblies. As the order of the genes in the plastomes and the placement of IRs was highly conserved in all assembled plastomes, we did not assemble the sequences of the remaining 20 species we sequenced.

The large copy number of plastomes per cell that we observed is not uncommon in Apocynaceae. Similarly high proportions of plastome reads (11.8%) has been reported in *Asclepias*^11^. This is much higher than in most angiosperms, where c. 1% of sequencing reads mapping to plastomes is common (pers. obs.). The high copy number helps in part to reduce assembly issues derived from the highly repetitive intergenic parts of the *Hoya* plastome; however, routine assembly of *Hoya* plastomes from short sequencing reads is likely to remain challenging. Even with the very high coverage that we attained, some low GC content intergenic regions had very low or even zero sequence coverage, leading to gaps in some of our assemblies. We think this was likely due to heavy degradation of the plastome, which may have occurred as leaves age. Use of younger leaf tissue might help to avoid this issue.

The assembly of the mitogenome was even more time consuming, as iterative extension of sequences to bridge gaps was soon interrupted by presence of plastome sequences. Assembling reads from other species to the already assembled mitogenome of *H*. *lithophytica* did not help much, suggesting that there is a high level of instability of mitogenomes within *Hoya*.

The gene order and overall architecture of the ingroup samples is highly similar to that reported by Wei et al.^26^ in *Hoya carnosa*, but all our assemblies differed significantly from those reported by Tan et al.^25^ in *H*. *verticillata* and *H*. *diversifolia*. We have reported CDSs and/or exons omitted by Wei et al.^26^, specifically *accD*, *ndhD*, *ndhH*, *ycf2* and *ycf15*, as a corresponding open reading frame was present. Tan et al.^25^ also omitted the CDSs of *accD*, *ndhH*, *ycf1*, and *ycf2*.

The dramatic IR–LSC boundary shift reported by Wei et al.^26^ is shared by the entire *Hoya* group, including *Papuahoya*, whose plastome is strongly supported to have diverged before all other ingroup taxa. Since all ingroup taxa included this boundary change which is not seen in any of the outgroups used, it can be considered a synapomorphy for the *Hoya* group.

The boundary shift was not reported by Tan et al.^25^, but we believe that this was in error. Our study includes conspecific sequences to those they reported, and in our analyses, they clearly shared the structure with the other *Hoya* group species. The two species are deeply nested in the phylogeny of the *Hoya* group.

The genome structure of *Hoya* has some parallels to other Asclepiads. As reported for *Asclepias*^13^ the intergenic regions of *Hoya* have long repeated regions of very low GC content. These regions make it difficult to map reads of *Hoya* even to a closely related species, and undoubtedly offer a challenge in use of intergenic reads. However, the boundary-shift observed is unique to the *Hoya* group.

Outside of Apocynaceae, there are clear parallels between the plastome restructuring in the *Hoya* group and that of *Lamprocapnos spectabilis* (L.) Fukuhara (Papaveraceae). Park et al.^29^ reported the extension of the IR/SSC boundary from *ycf1* (outgroup) to *ndhF*, and *Lamprocapnos* also has AARs in *accD* and *ycf1* (however, this was not mentioned in *ycf2*). Unlike in *Lamprocapnos*, no additional inversions or other changes to gene order were seen in our study in relation to the outgroup used. The SSC observed in *Lamprocapnos* is the smallest known in any plant, but only slightly smaller than that observed in species in the *Hoya* group.

The phylogenetic tree obtained is congruent with the most recent phylogeny of the *Hoya* group^7^, with the recognition of the monophyletic genera *Papuahoya*, *Oreosparte* and *Dischidia* that are fully supported.

Based on Rodda et al.^7^ the relationships between *Oreosparte*, *Dischidia* and *Hoya* as well as Group 1 and numerous smaller clades within *Hoya* are not fully supported (their Fig. 4). Our analysis (Fig. 6) confirms that the earliest divergent genus in the *Hoya* group is *Papuahoya*, followed by *Dischidia*. *Dischidia* is sister to a clade which is not fully supported (77% BS, 1 BPP) including all species currently attributed to *Hoya* and *Oreosparte*. Species of *Hoya* were segregated in two clades in Rodda et al.^7^, one (their Clade I) including four species from continental Asia (100% BS) the other containing the rest of the species (80% BS). Basal clades in Group 1 of *Hoya* were unsupported (53–69% BS, their Fig. 4). In our analysis instead their Clade I is deeply nested in *Hoya s*.*s.*, while *Hoya* is still separated into two clades, the first (Clade II) including species formerly included in *Eriostemma* + *Clemensiella* (100% BS, 1 BPP) is sister to *Oreosparte* + *Hoya s.s.*, the second (*Hoya s.s.*, 99% BS, 1 BPP) is sister to *Oreosparte*. Clade II can therefore be tentatively classified under the already available genus name *Clemensiella*, here represented by *C*. *omlorii*. *Hoya coronaria* and *H*. *gigas* have also been alternatively classified in the genus *Eriostemma* (type species: *Hoya coronaria*), which could now be considered a synonym of *Clemensiella*. This clade also includes *H*. *lithophytica*, a rock dwelling species from NW Thailand. The four species in this clade are characterised by terrestrial (or hemi epiphytic) climbing habit and by having pollinia without pellucid margins. These characters are unique to this clade among the species we sampled here, but not unique in the genus as other species can be terrestrial and lack pellucid margins of the pollinium (e.g. *Hoya surisana* Rodda & S.Rahayu). The second *Hoya* clade that includes the type of the genus *H*. *carnosa* is to be considered as *Hoya s*.*s*.

Based on our results either *Hoya* needs to be separated in two genera, *Hoya* and *Clemensiella*, or *Hoya* needs to be more broadly circumscribed to also include species currently in *Oreosparte*. In this latter scenario *Clemensiella*, *Oreosparte* and Clade I (Group 1 and 2) of *Hoya* may be allocated to subgeneric rank.

Our sampling of the *Clemensiella* clade is limited and samples of more taxa are needed to verify whether *Clemensiella* and *Eriostemma* should be kept separated (either at generic or subgeneric level).

Before making any nomenclatural changes, a more extensive phylogeny should be generated including extensive nuclear data to verify that the topology is congruent and that the observed clades are supported.

## Materials and methods

We sequenced 38 species in the *Hoya* group, and three outgroups (in *Marsdenia* R.Br. *s*.*l.*, and *Jasminanthes* Blume). Outgroups were selected due to their known position as outgroups of *Hoya* group (Rodda et al.^7^). The ingroups were selected to represent all the genera of the *Hoya* group where material is available (*Hoya*, *Dischidia*, *Oreosparte*, *Papuahoya*). Within *Hoya* we included at least one sample for each of the main *Hoya* clades (clades I to VI) of Wanntorp et al.^10^ and Rodda et al.^7^. For *Dischidia* we included eight taxa that represent the morphological variation of the genus, including *Dischidia parasita* (Blanco) Arshed, Agoo & Rodda, the type of the synonymous genus *Dischidiopsis* Schltr. *Oreosparte* and *Papuahoya* are represented by two species each.

### Plant materials and DNA extraction

The leaves were collected from plants cultivated at the Singapore Botanic Gardens or obtained from herbarium specimens. All plant specimens used for this study were collected to the best of our knowledge in compliance with local, institutional, national, or international regulations at the time of collection. All newly prepared voucher specimens were deposited in the Singapore Botanic Gardens Herbarium (SING). Their information is summarised in Table 2. The herbaria acronyms follow Thiers^31^.

**Table 2:** Sampled taxa used in this study: voucher specimens, GenBank, BioProject and BioSample accession numbers.

Taxon	Herbarium	Voucher	Geographic origin	GenBank Accession numbers plastome/mitogenome	BioProject/BioSample accession numbers
*Dischidia acutifolia*	SING	Rodda MR898	Singapore, cultivated Singapore Botanic Gardens	MW719066/–	PRJNA706870/SAMN18147001
*Dischidia albida*	A	Middleton et al. 3050	Thailand	MG963260/–	–/–
*Dischidia hirsuta*	SING	Rodda et al. MR11-008	Singapore	–/–	PRJNA706870/SAMN18147002
*Dischidia milnei*	SING	Rodda MR12-H211	Papua New Guinea, cultivated Singapore Botanic Gardens	MW719059/–	PRJNA706870/SAMN18147003
*Dischidia nummularia*	SING	Rodda MR313	Singapore	MW719070/–	PRJNA706870/SAMN18147004
*Dischidia ovata*	SING	Rodda MR2095	Australia, cultivated Singapore Botanic Gardens	–/–	PRJNA706870/SAMN18147005
*Dischidia parasita*	SING	Rodda MR507	Philippines, cultivated in Singapore, Gardens by the Bay	MW719057/–	PRJNA706870/SAMN18147006
*Dischidia rimicola*	SING	Rodda MR543	cultivated Singapore Botanic Gardens	–/–	PRJNA706870/SAMN18147007
*Hoya bakoensis*	SING	Rodda MR1042b	Malaysia, Sarawak	–/–	PRJNA706870/SAMN18147008
*Hoya carnosa*	not specified	not specified	China	NC_045868/–	–/–
*Hoya coronaria*	SING	Rodda MR333	Singapore, cultivated Singapore Botanic Gardens	MW719064/–	PRJNA706870/SAMN18147009
*Hoya diversifolia*	SING	Rodda MR12-S040	Singapore	MW719073/–	PRJNA706870/SAMN18147010
*Hoya diversifolia* (*liangii*)	not specified	not specified	China	NC_042245/–	–/–
*Hoya exilis*	SING	Rodda MR731	Papua New Guinea, cultivated Singapore Botanic Gardens	MW719054/–	PRJNA706870/SAMN18147011
*Hoya gigas*	SING	Nyhuus s.n	Papua New Guinea, cultivated Uppsala Botanic Garden	–/–	PRJNA706870/SAMN18147012
*Hoya hamiltoniorum*	SAN	Gokusing & Lombika in Lamb 1814/2009	Borneo, Malaysia, Sabah	MW719068/–	PRJNA706870/SAMN18147013
*Hoya ignorata*	SING	Simonsson & Somadee NS10-004 (SING)	Thailand	MW719061/–	PRJNA706870/SAMN18147014
*Hoya imperialis*	SING	Rodda MR340	Cultivated Singapore Botanic Gardens	–/–	PRJNA706870/SAMN18147015
*Hoya inconspicua*	SING	Rodda MR913	Papua New Guinea, cultivated Singapore Botanic Gardens	–/–	PRJNA706870/SAMN18147016
*Hoya inflata*	BRI	Liddle IML1076	Papua New Guinea	–/–	PRJNA706870/SAMN18147017
*Hoya insularis*	SING	Somadee s.n	Borneo, cultivated Thailand	MW719067/–	PRJNA706870/SAMN18147018
*Hoya lanceolata*	SING	Rodda MR1769	Nepal, cultivated Singapore Botanic Gardens	–/–	PRJNA706870/SAMN18147019
*Hoya latifolia*	SING	Rodda MR1115	Singapore, cultivated Singapore Botanic Gardens	MW719069/–	PRJNA706870/SAMN18147020
*Hoya lithophytica*	SING	Nyhuus s.n	Thailand, cultivated Sweden	MW719058/MW719051	PRJNA706870/SAMN18147021
*Hoya lyi*	SING	Rodda M. MR542	Laos, cultivated Singapore Botanic Gardens	MW719055/–	PRJNA706870/SAMN18147022
*Hoya megalaster*	SING	Rodda MR746	Papua New guinea, Madang, Cultivated in Thailand, Chonburi, Nong Nooch Tropical Garden	MW719063/–	PRJNA706870/SAMN18147023
*Hoya monetteae*	SAN	Linus Gokusing in Lamb AL2321/2012	Malaysia, Sabah, Cultivated at Kipandi Park	MW719053/–	PRJNA706870/SAMN18147024
*Hoya nicholsoniae*	SING	Rodda MR718	Papua New Guinea, cultivated Singapore Botanic Gardens	–/–	PRJNA706870/SAMN18147025
*Hoya obtusifoli*a	SING	Lai SING2016-165	Singapore	–/–	PRJNA706870/SAMN18147026
*Hoya omlorii*	SING	Rodda MR304	Malaysia, Perak, cultivated Singapore Botanic Gardens	MW719060/–	PRJNA706870/SAMN18147027
*Hoya platycaulis*	K	s.coll., s.n	Philippines, Laguna	–/–	PRJNA706870/SAMN18147028
*Hoya scortechinii*	SING	Rodda MR711	Singapore, cultivated Singapore Botanic Gardens	–/–	PRJNA706870/SAMN18147029
*Hoya thailandica*	BRI	Liddle IML1493	Thailand, cultivated Australia	MW719072/–	PRJNA706870/SAMN18147030
*Hoya undulata*	SING	Rodda MR650	cultivated Singapore Botanic Gardens	–/–	PRJNA706870/SAMN18147031
*Hoya verticillata*	SING	Rodda MR1030	Singapore, cultivated Singapore Botanic Gardens	MW719071/–	PRJNA706870/SAMN18147032
*Hoya verticillata*	not specified	not specified	China	NC_042246/–	–/–
*Hoya wallichii*	SING	Rodda MR1825	Cultivated Singapore Botanic Gardens	–/–	PRJNA706870/SAMN18147033
*Hoya walliniana*	SING	Rodda MR1044A	Malaysia, Sarawak	–/–	PRJNA706870/SAMN18147034
*Jasminanthes maingayi*	SING	Rodda MR691	Singapore	MW719056/–	PRJNA706870/SAMN18147035
*Marsdenia flavescens*	BRI	Forster 28,686	Australia	MW719052/–	PRJNA706870/SAMN18147036
*Marsdenia longipedicellata*	A	Gray 7487	Australia	–/–	PRJNA706870/SAMN18147037
*Oreosparte celebica*	E	Middleton 3700	Indonesia, Sulawesi, cultivated Royal Botanic Garden Edinburgh	MW719065/–	PRJNA706870/SAMN18147038
*Oreosparte parviflora*	SING	Rodda MR1786	Cultivated Singapore Botanic Gardens	–/–	PRJNA706870/SAMN18147039
*Papuahoya neoguineensis*	SING	Rodda MR1116	Papua New Guinea, cultivated Singapore Botanic Gardens	–/–	PRJNA706870/SAMN18147040
*Papuahoya urniflora*	SING	Simonsson Juhonewe & Juhonewe NS0069L	Papua New Guinea	MW719062/–	PRJNA706870/SAMN18147041

Fresh, silica-dried or herbarium leaf samples were extracted using DNeasy Plant Mini Kit (Qiagen Inc., Valencia, California, U.S.A.). A minimum of 400 ng of total genomic DNA was sent for library preparation and genome skimming sequencing using Illumina HiSeq (AITbiosciences, Singapore). A minimum of 1 Gbp of sequence with a read length of 100 bp were acquired per sample. Sequence quality filtering was done with Geneious 11.1.2 (Biomatters Ltd, New Zealand) trim and filter function, using error probability limit of 0.05, a minimum read length of 70 and removing adapters with a minimum blast alignment score of 16.

### Sequencing, assembly and annotation

The plastome of *Hoya lithophytica* was assembled first, using a combination of GetOrganelle^32^, and assemblies to several reference genomes in Geneious 11.1.2 and ORG.asm^33^ without a reference or using a variety of Apocynaceae plastomes as reference. The automated assemblies had a large number of artefacts, mostly due to frequent gene movement between the plastome and the mitogenome. The assemblies were checked by assembling sequencing reads to the initial assembled genomes in Geneious 11.1.2, followed by visual correction of alignment, and extension of gaps using iterative assemblies. The approximate length of the inverted repeat was estimated by observing the part of the genome with high sequencing coverage, and areas of exceptionally low coverage were identified as mitogenome sequences; the position of the inverted repeats was approximately corrected, the mitogenome reads were removed from the assemblies, and further iterative gap filling was carried out, resulting in a full circular plastome.

Twenty one plastomes (19 in the *Hoya* group and two outgroup taxa) were assembled to *Hoya lithophytica* in Geneious 11.1.2, with a manual correction of alignment and gaps (with iterative extension of gaps when required), followed by further assemblies to the resulting genome to detect and correct errors. In a few cases, one difficult to sequence region (intergenic region *psbA–trnH*) was acquired through Sanger Sequencing (AITbiosciences, Singapore). Other gaps were not corrected if present.

For 20 further species (19 in the *Hoya* group and one outgroup taxon), the assembly of the entire plastome was not attempted, and only exons were assembled by aligning them to the reference.

Final circular plastomes were checked by re-mapping the filtered reads to the plastome using Geneious 11.1.2 (Biomatters Ltd, New Zealand) read mapper using low sensitivity, adjusted to not allowing gaps, and alignments were visually inspected for errors and gaps.

Sequences were annotated by transferring annotation from the published plastome of *Hoya liangii* (a synonym of *Hoya diversifolia*) (GenBank accession number NC_042245^25^), followed by correction of position of CDSs. Sliding window analysis was conducted to generate the nucleotide diversity (Pi) of complete ingroup plastomes. The plastomes were aligned using MAFFT v.7.309^34^, using scoring matrix 200PAM / k = 2, gap open penalty of 1.53 and offset value of 0.123. The resulting alignment was analysed using DnaSP v. 6.12.03^35^ to compare levels of nucleotide variation across the plastomes.

The mitogenome of *Hoya lithophytica* was constructed by filtering sequence reads that completely matched the plastome, and mapping the remaining reads to the mitogenome of *Asclepias syriaca* (KF541337). While parts of the mitogenome were identical to the plastome, enough reads with a single read error were present to cover all parts of the mitogenome for unambiguous assembly. Only small fragments initially matched the reference genome. The other parts of the mitogenome were assembled by iterative mapping and by identifying the boundaries of mitogenome/plastome overlap by mapping reads to the plastome. Attempts to construct further mitogenomes were abandoned once we identified massive restructuring of the mitogenome even within the ingroup.

Gene movement from plastome to the mitogenome was estimated by cutting the plastome of *Hoya lithophytica* into 30 bp fragments, and measuring the percentage of resulting fragments that mapped to the mitogenome, using the Geneious mapper in Geneious 11.1.2 (Biomatters Ltd, New Zealand).

Changes in plastomes organisation were compared between major clades in the ingroup and the outgroups as well as published plastomes representing a variety of informal groups of Apocynaceae. The SSC was arranged in the same direction, one of the inverted repeats was removed, and the plastomes were analysed using progessiveMauve in Mauve 2015-02-25^36^. The following plastomes from GenBank were used in the comparison: *Rhazya stricta* Decne. (KJ123753), *Carissa macrocarpa* (Eckl.) A.DC. (NC_033354), *Trachelospermum jasminoides* (Lindl.) Lem. (MK783315), *Cynanchum wilfordii* (Maxim.) Hook.f. (KT220733), *Asclepias syriaca* (NC_022432).

### Phylogenetic analysis

For phylogenetic analyses, all exons over 90 bp were extracted from the 41 newly sequenced samples as well as the three *Hoya* and one *Dischidia* plastomes available in GenBank^16,25,26^ and aligned using setting-auto in mafft^34^, and alignments were checked using setting -automated1 in trimAl^37^. Shorter exons could not be reliably retrieved for the species for which we did not have a complete plastome. Three protein coding genes (*accD*, *ycf1* and *ycf2*, all one-exon genes) had long amino acid repeats that could not be aligned unambiguously. These were removed from the phylogenetic analyses. Removing areas with gaps in alignment did not affect the phylogeny or branch support noticeably, and these areas were retained in the final phylogenetic analysis. The exon alignments were partitioned to one exon per partition. A maximum likelihood tree was generated using IQ-TREE 2.0.6^38^, with an independent substitution model test (ModelTest) for each partition. The settings for the maximum likelihood analyses were: -m MFP + MERGE -T 12 with 1000 bootstrap replicates. The models selected were: TVM + F + R3 (*atpB*, *infA*, *petN*, *atpE*, *ndhH*, *rpoA*, *psbH*, *rpl14*, *rpoB*, *atpF*, *ndhJ*, *rpl16*, *rpoC1*, *rps2*, *trnS*-*UGA*, *ndhK*, *psaI*, *rpoC1*, *atpI*, *petA*, *psaJ*, *rpoC2*, *rps11*, *rps4*, *ycf3*, *psbN*, *rps14*, *rps8*), TVM + F + R4 (*clpP*, *ndhF*, *ycf4*, *clpP*), K3Pu + F + I (*psbD*, *ndhI*, *psbI*, *ycf3*, *psbK*, *ycf3*, *petB*, *rpl23*, *petD*, *psbB*, *rpl2*, *rps12*, *rps7*, *petG*, *psbC*, *rpl2*), K3Pu + F + R2 (*psbT*, *rps15*, *rps18*, *rps36*, *ccsA*), K3Pu + F + R2 (*rpl32*, *ndhG*, *psbE*, *rpl33*, *psaA*, *psbF*, *rbcL*, *atpF*, *ndhA*, *psaB*, *rps19*, *ndhA*, *psaC*, *atpH*, *ndhB*, *psbJ*, *ndhB*, *ndhC*, *psbA*, *psbL*, *ndhD*, *ndhE*), HKY + F + I (*rrn16*, *rrn23*, *rrn4*.5, *rrn5*) and K3Pu + F + R2 (*psbZ*, *rps16*, *matK*, *rpl20*, *rpl22*, *rps3*, *cemA*, *psbM*). For some genes there was more than one exon^39^. *Jasminanthes maingayi* (Hook.f.) Rodda and *Marsdenia longipedicellata* P.I.Forst. were selected as the outgroup, as they were known to be part of a clade that is sister to the other species included.

Baysian support for the nodes was tested with MrBayes 3.2.5^40^. We used 30,000,000 Markov chain Monte Carlo iterations, keeping one tree every 100 generations, with a burn-in of 25% (mcmc ngen = 30,000,000 samplefreq = 100 burnin = 75,000). We used the exon-partitioned sequence alignments generated for the IQ-TREE 2.0.6^38^, and applied the same model, GTR + Gamma (lset nst = 6 rates = gamma) for all partitions, with rates unlinked between datasets (unlink statefreq = (all) revmat = (all) shape = (all) pinvar = (all), prset applyto = (all) ratepr = variable). We accepted the results if the likelihoods had converged and the minimum estimated sample size was over 100 for all parameters by the end of the run. To test the effect of the model used, we also ran the same analysis with all substitution rates set to equal and equal rate variation (lset nst = 1 rates = equal).

### Data archiving statement

Raw, demultiplexed sequence reads are available at the Sequence Read Archive (https://www.ncbi.nlm.nih.gov/sra) and can be accessed with the BioProject IDs listed in Table 2. The complete or incomplete plastome sequence data of the 38 species sequenced as well as the complete mitochondrial genome of *H*. *lithophytica* obtained for this study have been deposited to the GenBank of NCBI (see Table 2 for accession numbers). The sequence alignment is available in Figshare at https://doi.org/10.6084/m9.figshare.14189021.
